# Radiomics Based on Contrast-Enhanced MRI in Differentiation Between Fat-Poor Angiomyolipoma and Hepatocellular Carcinoma in Noncirrhotic Liver: A Multicenter Analysis

**DOI:** 10.3389/fonc.2021.744756

**Published:** 2021-10-13

**Authors:** Xiangtian Zhao, Yukun Zhou, Yuan Zhang, Lujun Han, Li Mao, Yizhou Yu, Xiuli Li, Mengsu Zeng, Mingliang Wang, Zaiyi Liu

**Affiliations:** ^1^ Department of Radiology, Guangdong Provincial People’s Hospital, Guangdong Academy of Medical Sciences, Guangzhou, China; ^2^ Medical Imaging Center, The First Affiliated Hospital of Guangzhou University of Chinese Medicine, Guangzhou, China; ^3^ Department of Radiology, Sun Yat-Sen University Cancer Center, Guangzhou, China; ^4^ AI Lab, Deepwise Healthcare, Beijing, China; ^5^ Department of Radiology, Zhongshan Hospital, Fudan University, Shanghai, China

**Keywords:** hepatocellular carcinoma, angiomyolipoma, radiomics, magnetic resonance imaging, machine learning

## Abstract

**Objective:**

This study aims to develop and externally validate a contrast-enhanced magnetic resonance imaging (CE-MRI) radiomics-based model for preoperative differentiation between fat-poor angiomyolipoma (fp-AML) and hepatocellular carcinoma (HCC) in patients with noncirrhotic livers and to compare the diagnostic performance with that of two radiologists.

**Methods:**

This retrospective study was performed with 165 patients with noncirrhotic livers from three medical centers. The dataset was divided into a training cohort (*n* = 99), a time-independent internal validation cohort (*n* = 24) from one center, and an external validation cohort (*n* = 42) from the remaining two centers. The volumes of interest were contoured on the arterial phase (AP) images and then registered to the venous phase (VP) and delayed phase (DP), and a total of 3,396 radiomics features were extracted from the three phases. After the joint mutual information maximization feature selection procedure, four radiomics logistic regression classifiers, including the AP model, VP model, DP model, and combined model, were built. The area under the receiver operating characteristic curve (AUC), diagnostic accuracy, sensitivity, and specificity of each radiomics model and those of two radiologists were evaluated and compared.

**Results:**

The AUCs of the combined model reached 0.789 (95%CI, 0.579–0.999) in the internal validation cohort and 0.730 (95%CI, 0.563–0.896) in the external validation cohort, higher than the AP model (AUCs, 0.711 and 0.638) and significantly higher than the VP model (AUCs, 0.594 and 0.610) and the DP model (AUCs, 0.547 and 0.538). The diagnostic accuracy, sensitivity, and specificity of the combined model were 0.708, 0.625, and 0.750 in the internal validation cohort and 0.619, 0.786, and 0.536 in the external validation cohort, respectively. The AUCs for the two radiologists were 0.656 and 0.594 in the internal validation cohort and 0.643 and 0.500 in the external validation cohort. The AUCs of the combined model surpassed those of the two radiologists and were significantly higher than that of the junior one in both validation cohorts.

**Conclusions:**

The proposed radiomics model based on triple-phase CE-MRI images was proven to be useful for differentiating between fp-AML and HCC and yielded comparable or better performance than two radiologists in different centers, with different scanners and different scanning parameters.

## Introduction

Hepatic angiomyolipoma (AML) is a mesenchymal benign tumor belonging to the perivascular epithelioid cell tumors (PEComas), which is a group of tumors believed to be derived from perivascular epithelioid cells and the co-expression of melanocytic and muscle marker. Histologically, it contains variable proportions of blood vessels, smooth muscle cells, and adipose tissue. Although only a few hundred cases of hepatic AMLs have ever been recorded all over the world, increasing numbers of cases are being reported due to the development of modern imaging techniques in recent years (1). The hepatic AML lesions often grow slowly and do not cause any clinical symptoms. Therefore, once the diagnosis of AML is established, conservative treatment and annual imaging follow-up is recommended in patients without indications for surgical resection (2). Typically, the diagnosis of AML is suggested in the case of a middle-aged woman when a solitary tumor occurs in a noncirrhotic liver and intratumoral macroscopic fat is detected on computed tomography (CT) or magnetic resonance imaging (MRI) (3). However, the amount of fat component in the hepatic AML varies greatly, ranging from 10 to 90% of the tumor volume (4) and, in some instances, cannot be easily identified on imaging (5, 6). In that case, many radiologists tend to misdiagnose these fat-poor AMLs (fp-AMLs) as other common hypervascular liver tumors, particularly hepatocellular carcinoma (HCC), with a frequency of 50% due to the overlapping imaging features (7), especially in areas with a high prevalence of hepatic viral infections like China. This can lead to unsuitable therapeutic schemes such as surgical therapy and liver transplantation. Therefore, it is crucial to accurately distinguish between fp-AML and HCC before surgery.

Unfortunately, correct preoperative diagnosis of fp-AML is currently challenging and mainly depends on histological findings. It is well known that a clinical history of chronic liver disease may be an important clue for the diagnosis of HCC, such as cirrhosis caused by hepatitis B virus (HBV) or hepatitis C virus (HCV) or excessive alcohol use. However, up to 20–30% of HCCs can develop in patients with normal livers (8). Hepatic AML has also been reported to occur in hepatitis B carriers (9). In terms of imaging, it has been proven that it is difficult to differentiate fp-AML from HCC in noncirrhotic liver by the use of only a dynamic enhancement pattern as most of the tumors are seen as a well-defined, hypervascular enhancing mass on arterial phase (AP), followed by a washout pattern on venous phase (VP) or equilibrium phase (6). Besides this, although previous studies pointed out that the presence of early draining vein and absent tumor capsule were useful findings for the differentiation of fp-AML from HCC in noncirrhotic liver (6, 10), these signs were subjective and dependent on the experience of the radiologist (5). In addition, preoperative fine needle aspiration cytology (FNAC) of AML can obtain definite histological evidence to improve the diagnostic accuracy with negligible risk (11). However, FNAC has some limitations because the trabecular growth pattern in hepatic epithelioid AML may mimic the cells of HCC (12).

Radiomics is an emerging field in image analysis, which extracts a large number of high-dimensional quantitative features from the image data and provides information that reflects the underlying pathophysiology (13). Several studies have proven that MRI-based radiomics features have the ability to discriminate different tumor phenotypes (14–17). We assumed that, using radiomics, we could extract and quantify the differences in conventional contrast-enhanced MRI (CE-MRI) images between fp-AML and HCC.

In this study, we aimed to develop a radiomics model based on triple-phase CE-MRI images to differentiate between fp-AML and HCC in the noncirrhotic liver and validate using external data. Moreover, we compared the diagnostic performance of radiomics model and radiologists in distinguishing these two kinds of tumors.

## Materials and Methods

### Patient Population

This multicenter retrospective study was carried out in three centers: Shanghai Zhongshan Hospital (center A), Guangdong Sun Yat-Sen University Cancer Center (center B), and Guangdong Provincial People’s Hospital (center C), approved by the institutional review board of each center, and patient informed consent was waived.

**Figure 1:**
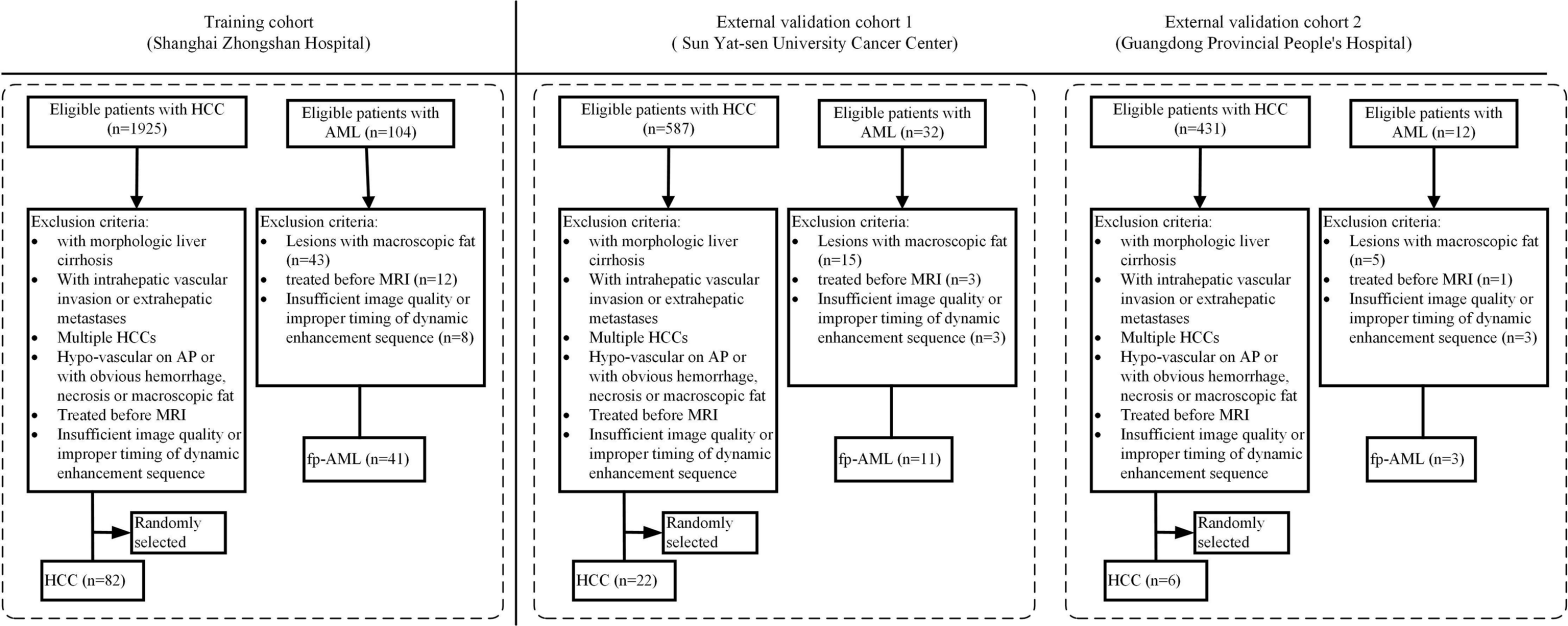
The patient enrollment process for this study.

The patient enrollment process for this study is shown in **Figure 1**. First, a thorough search of the electronic medical record system of each center was performed between January 2012 and December 2019 for the diagnosis of hepatic AML. All the patients who both had a histologic diagnosis of AML and had undergone a liver MRI using the contrast agent gadolinium-diethylene triamine pentaacetic acid (Gd-DTPA) within 15 days before their surgery were included. The exclusion criteria were as follows: (1) patients with the presence of macroscopic intralesional fat on unenhanced T1-weighted (T1W) images (lose signal at fat saturation imaging or demonstrate etching artifact at the fat–water interface at chemical shift imaging) (18), (2) patients who received chemotherapy or radiotherapy before surgery, and (3) patients with insufficient CE-MRI image quality or improper timing for dynamic enhancement sequence.

To establish a control group, we subsequently searched the same databases of each hospital for an initial diagnosis of HCC during the same period by applying the same inclusion criteria. The exclusion criteria were as follows: (1) lesions with obvious necrosis, cyst, hemorrhage, or macroscopic fat, (2) lesions with hypo-enhancement on AP, (3) patients who received chemotherapy or radiotherapy before surgery, (4) patients with multiple HCCs, (5) patients with insufficient CE-MRI image quality or improper timing for dynamic enhancement sequence, (6) lesions with intrahepatic vascular invasion or extrahepatic metastases, and (7) patients with morphologic liver cirrhosis (19). Consequently, the patients who had a single and hypervascular HCC without definite evidence of morphologic cirrhosis were identified in each center. In view of the fact that AMLs are much less common than HCCs, we randomly selected some of these patients according to the ratio of 1:2 to alleviate the offset caused by the distribution and improve the statistical power (20), relative to the number of AML patients who were eventually enrolled in each center, using a commercially available random number generator (QuickCalcs, GraphPad).

In total, 165 patients were enrolled in this multicenter study, including 55 fp-AMLs (center A, *n* = 41; center B, *n* = 11; and center C, *n* = 3) and 110 HCCs (center A, *n* = 82; center B, *n* = 22; and center C, *n* = 6). Considering the small sample sizes of center B and center C, we grouped the patients from these two centers into one external validation cohort.

For center A, according to the TRIPOD statement, the patients were divided into training and internal validation cohorts according to the time of receiving surgical treatment and the ratio of 4:1. A total of 99 patients treated between February 2012 and January 2017 constituted the training cohort, whereas 24 patients treated between March 2017 and December 2019 constituted the internal validation cohort.

### CE-MRI Image Acquisition

The MRI examinations were performed using 1.5- or 3.0-T systems from various vendors. At each center, the MRI protocols contained unenhanced images and dynamic sequences after an intravenous contrast agent injection, including axial fat saturation (fs) T2-weighted (T2W), T1W in-phase/out-of-phase, unenhanced axial fs T1W and dynamic triple-phase CE-MRI. All patients received 0.2 mmol/kg body weight of Gd-DTPA (Magnevist, Bayer Schering Pharma, Berlin, Germany) *via* a power injector (Spectris Solaris^®^ EP MR, MEDRAD Inc., Indianola, IA, USA) at an infusion rate of 1.5–2 ml/s. After an intravenous contrast agent injection, three-dimensional fs T1W gradient-echo sequence [VIBE (Siemens Healthcare), LAVA (GE Healthcare), and THRIVE (Philips Healthcare)] was used to acquire dynamic enhanced images. The images in AP, VP, and delayed phase (DP) were acquired during suspended respiration at 25–35, 60–75, and 150–180 s, respectively. The detailed parameters of CE-MRI sequences used in each imaging center are reported in **Table 1**.

**Table 1:** Detailed parameters of contrast-enhanced three-dimensional fs T1W gradient-echo sequences in each center.

Center	Scanner	Vendor	Field strength (t)	Patients	TR/TE (ms)	Matrix	Flip angle
Center A (*n* = 123)	Aera	Siemens	1.5	18	3.51/1.39	260 × 352	10°
	Avanto	Siemens	1.5	21	5.04/2.31	200 × 288	10°
	Ingenia	Philips	3.0	3	4.30/1.65	528 × 528	10°
	UIHMR560	UI	1.5	36	4.4/2.2	320 × 512	10°
	UIHMR770	UI	3.0	31	3.28/1.45	324 × 480	10°
	Verio	Siemens	3.0	14	4.07/1.43	250 × 352	9°
Center B (*n* = 33)	Achieva	Philips	3.0	3	3.12/1.51	480 × 480	10°
	Aera	Siemens	1.5	4	4.63/2.16	460 × 640	10°
	Discovery MR750	GE	3.0	4	4.05/1.64	512 × 512	15°
	Signa HDxt	GE	1.5	5	3.98/1.90	512 × 512	15°
	Trio	Siemens	3.0	10	4.15/1.86	250 × 320	9°
	uMR780	UI	3.0	7	3.3/1.45	336 × 480	10°
Center C (*n* = 9)	Ingenia	Philips	3.0	5	4.01/1.94	384 × 384	10°
	Achieva	Philips	3.0	4	4.02/1.94	384 × 384	10°

FS, fat-suppressed; UI, United Imaging; GE, General Electric; TE, echo time; T1W, T1-weighted; TR, repetition time.

### Radiologists Interpretation of the Enhanced MRI Images

Two abdominal radiologists (XZ and YZ, with 10 and 5 years of experience, respectively) independently reviewed the images of the internal and the external validation cohort. The radiologists were blinded to clinical information and did not know the exact number of each type of tumor but were aware that the tumors were finally diagnosed with fp-AML or HCC. The two radiologists assessed each specific phase and judged this based on the signal intensity of majority of the tumor. According to the features defined with reference to the definitions and annotations in the Liver Imaging Reporting and Data System (LI-RADS) (21), the main signs that were often used for differential diagnosis between fp-AML and HCC were recorded, including the draining hepatic vein and intra-tumor vessel, the presence of a complete capsule, and the pattern of enhancement (wash in and wash out or prolonged enhancement). When the lesion demonstrated specific MRI features such as intra-tumor vessel, draining hepatic vein, prolonged enhancement, no washout in the VP, and lack of complete capsule, it would be classified as fp-AML; otherwise, it would be classified as HCC (3, 10).

### Radiomics Workflow

**Figure 2:**
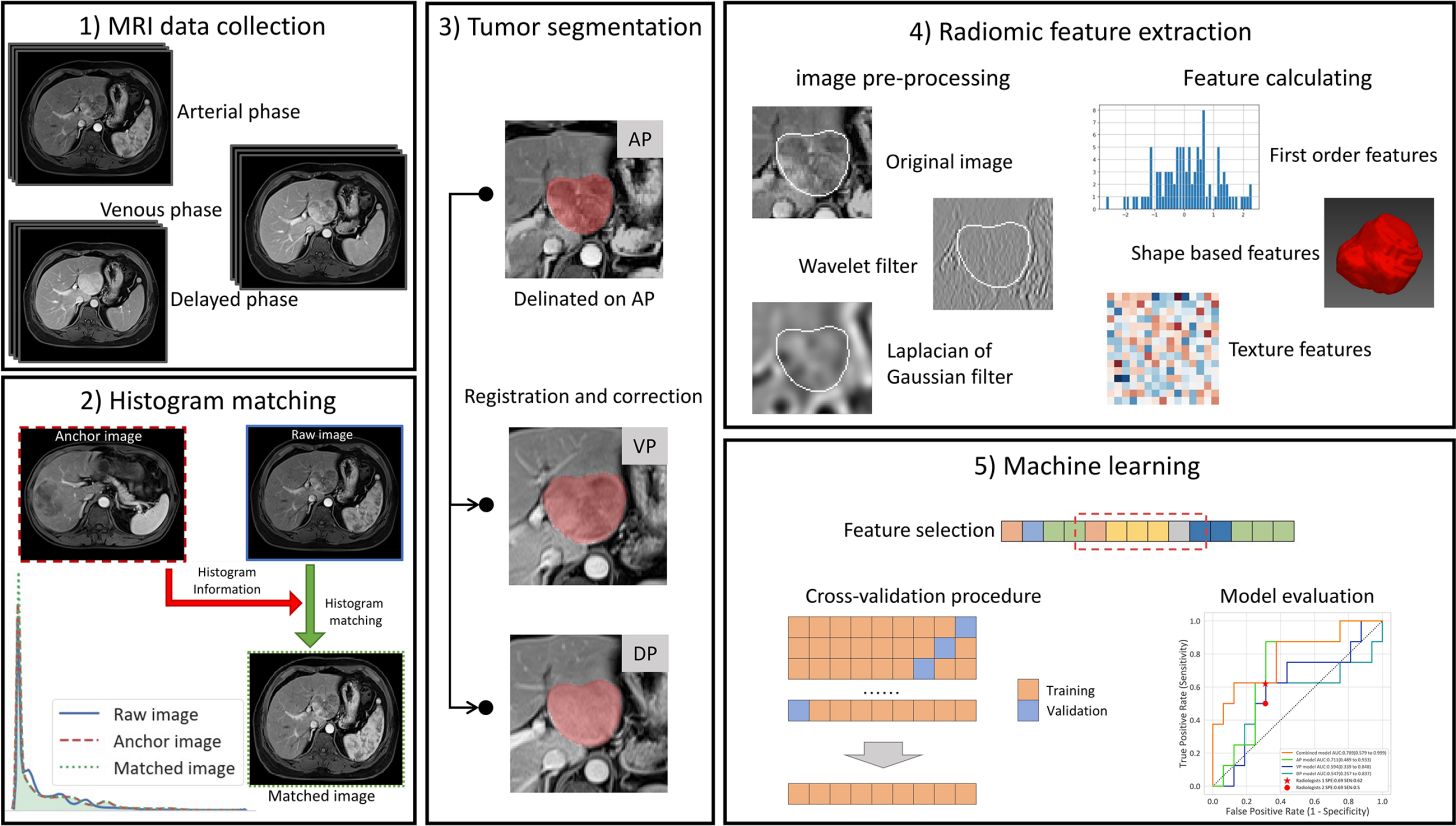
The workflow of our study. (1) The collection of CE-MRI data, including the arterial phase (AP), venous phase, and delayed phase images. (2) Histogram matching: The images of each phase were matched to the corresponding phase of the first patient by histogram matching. (3) Tumor segmentation: The delineation was performed on AP and then registered to the other two phases, and the misalignment was manually corrected. (4) Radiomic feature extraction: For each phase, the radiomic features were extracted from the tumor region of the original images and the preprocessed images. (5) Machine learning. The feature selection method was used to select the optimal feature subset, and then the models were trained by the cross-validation procedure and evaluated on the internal and external validation cohort.

An overview of our workflow is illustrated in **Figure 2**. Firstly, the enhanced MRI data were collected, including the AP, VP, and DP images. Then, the images of each phase were normalized by the histogram-matching method. The delineation was performed on AP and then registered to the other two phases, and the misalignment was manually corrected. For each phase, the radiomic features were extracted from the tumor region of the original images and the preprocessed images. Finally, the feature selection method was used to select the optimal feature subset. The models were trained by the cross-validation procedure and evaluated in the internal and external validation cohort.

### The Segmentation of Tumor Images

The tumor volume of interest (VOI) was manually delineated slice by slice using the Medical Imaging Interaction Toolkit (MITK) software (v.2013.12.00, Heidelberg, Germany), referencing to the sagittal and coronal images reconstructed by the software. To reduce the workload of segmentation and increase the accuracy of tumor contouring, differently from many previous studies, the manual VOI delineation was performed only on the AP images in our study, and delineation was registered to the DP and VP images by DEEDs, an efficient 3D discrete deformable alignment algorithm (22), in accordance with the image information of the three phases. It was proved that the DEEDs algorithm outperformed the other common registration algorithm and achieved a dice coefficient of 0.70 for the four large organs (liver, spleen, and kidneys) (23). Then, the misalignment between the image and the registered contour on the other two phases was manually corrected. In this way, when the part or whole of the tumor had a similar signal intensity to the surrounding liver parenchyma and it was difficult to manually outline the contour on a single phase, the VOI of AP could be used for reference, and the tumor contour could be relatively accurately confirmed under the condition of triple-phase image registration.

The inter-observer reliability and intra-observer reproducibility of feature extraction were tested using the inter- and intra-class correlation coefficients (ICCs). After 30 cases of CE-MRI images (10 fp-AMLs and 20 HCCs) were selected randomly, radiologist 1 (XZ) and radiologist 2 (YZ) performed VOI segmentation manually, respectively. Radiologist 2 repeated the VOI segmentation 2 weeks later to assess the intra-observer reproducibility. The feature extraction was considered to represent a good agreement when the ICC was greater than 0.8. The remaining image segmentation was performed by radiologist 2 and reviewed by radiologist 1.

### Radiomics Feature Extraction

In the case of MRI, the signal intensity values vary according to the acquisition parameters used, which affect the extracted radiomic features (24). To calibrate the variations due to the scanner manufacturer and magnetic field strength in our cohort, histogram standardization (25) was used to match the input image histogram onto the standard image (in our case, the MRI of the first patient in the training cohort).

Radiomics extraction was performed using Pyradiomics V2.1.0. The images were resampled to a pixel spacing of 1 × 1 × 1 mm to counteract the interference caused by the non-uniform spatial resolution. Then, the original images were preprocessed by the wavelet filters or Laplacian of Gaussian filters with different parameters. For each phase, 1,132 radiomic features were obtained from the original images and the preprocessed images: (1) 234 first-order features, (2) 14 shape-based features, (3) 286 gray-level co-occurrence matrix features (GLCM), (4) 208 gray-level size zone matrix features (GLSZM), (5) 208 gray-level run length matrix features, and (6) 182 gray-level dependence matrix features (GLDM). Finally, a total of 3,396 radiomic features were extracted from triple-phase CE-MRI images for each patient.

### Construction and Validation of the Radiomics Signatures

The radiomic features extracted from the AP, VP, and DP images were used to build the AP model, VP model, and DP model, respectively. Then, the combined model was trained on all radiomic features of the images of three phases. The construction strategies of the four models were the same.

The features were normalized by Z-score normalization before the model building. To avoid information disclosure, the mean and standard deviation values were calculated only on the training set, and the entire dataset was normalized by the mean and standard deviation values from the training set. The features with poor consistency (intra-ICC or inter-ICC lower than 0.8) were filtered out. To reduce the redundancy of the features and to avoid overfitting, the joint mutual information maximization (JMIM) method (25), which utilizes mutual information and the maximum–minimum criterion, was used to select the subset of features. Considering the sample size of the training cohort, 10 radiomic features (10% of the sample size of the training cohort) were selected to avoid over-fitting (26). The logistic regression (LR) model was built by a repetitive (five runs) 10-fold cross-validation using the training cohort. After the hyper-parameters were determined by the cross-validation procedure, the LR model with optimal parameters was built on the entire training cohort.

The area under the receiver operator characteristic (ROC) curve was used to evaluate the performance of the radiomic models. After the cutoff value that maximizes the Youden Index was obtained on the cross-validation result, the accuracy, sensitivity, and specificity were also calculated. The output of the prediction was calibrated by the isotonic regression method.

### Statistical Analysis

The ROC curves were drawn by using Matplotlib (version 3.1.0), and the area under the ROC curve (AUC), accuracy, sensitivity, and specificity were calculated by the Scikit-learn python package (version 0.20.3). The kappa consistency test was adopted to assess inter-observer agreement between the two radiologists. The level of agreement was interpreted as slight if *κ* was 0.01 to 0.20, fair if 0.21 to 0.40, moderate if 0.41 to 0.60, substantial if 0.61 to 0.80, and almost perfect if 0.81 to 1. The DeLong test was used for pairwise comparisons between the combined model and the remaining three models and between the best-performing radiomics model and each radiologist. For the comparison of the sensitivity and specificity between the best-performing radiomics model and the assessment of the radiologists, the McNemar chi-square test was employed. The abovementioned statistical analysis was performed on R software (version 3.6.0; https://www.r-project.org/) environments. A two-sided *p <*0.05 was considered statistically significant throughout the study.

## Results

### Patient Demographics

The mean age of patients in the fp-AML group was lower than that of patients in the HCC group (47.1 ± 12.6 *vs*. 55.8 ± 12.0 years, *t* = -4.306, *p* < 0.001). Male predominance was observed in the HCC group, while most fp-AML patients were female [87% (48/55) *vs*. 18% (20/110), *p* < 0.001].

There was no patient with tuberous sclerosis in the fp-AML group. In the HCC group, more patients had a preexisting chronic liver disease that was caused by chronic HBV or HCV infection, compared with those with fp-AML [79% (87/110) *vs*. 9% (5/55), *p* < 0.001). None of the HCCs was of the fibrolamellar variant.

### Radiomics Analysis

Of the 3,396 radiomics features extracted from AP, VP, and DP images, 2,585 were demonstrated to have a good inter- and intra-observer agreement, including 958 AP features, 823 VP features, and 804 DP features. Then, the JMIM feature selection method selected 10 optimal features for each model. For the combined model, the 10 optimal features included seven features from AP, two features from DP, and one feature from VP.

**Figure 3:**
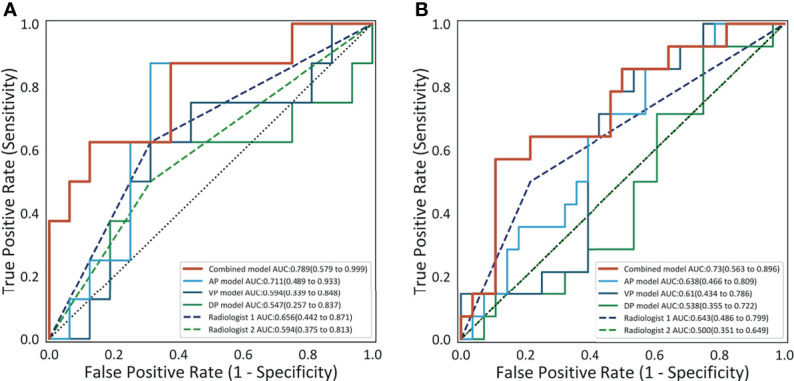
The receiver operating characteristic curves of the four models and the performance of the two radiologists on the internal validation cohort **(A)** and on the external validation cohort **(B)**.

The detailed diagnostic performance of each model is shown in **Table 2**. The AUCs of the AP model, the VP model, and the DP model reached 0.711 (95%CI, 0.489–0.933), 0.594 (95%CI, 0.339–0.848), and 0.547 (95%CI, 0.257–0.837) in the internal validation cohort and 0.638 (95%CI, 0.466–0.809), 0.61 (95%CI, 0.434–0.786), and 0.538 (95%CI, 0.355–0.722) in the external validation cohort. The combined model reached the AUC of 0.789 (95%CI, 0.579–0.999) in the internal validation cohort, significantly higher than the VP model (*p* = 0.015) and the DP model (*p* = 0.004). In the external validation cohort, the AUC of the combined model was 0.730 (95%CI, 0.563–0.896), also significantly higher than the VP model (*p* = 0.035) and the DP model (*p* = 0.008). The ROC curves are shown in **Figure 3**.

**Table 2:** The detailed performance of arterial phase (AP) model, venous phase (VP) model, delayed phase (DP) model, and combined model.

	Model	Area under the receiver operating characteristic curve (95%CI)	Accuracy	Sensitivity	Specificity
Training cohort (*n* = 99)	AP	0.863 (0.776–0.95)	0.798 (79/99)	0.848 (28/33)	0.773 (51/66)
	VP	0.756 (0.659–0.853)	0.636 (63/99)	0.879 (29/33)	0.515 (34/66)
	DP	0.752 (0.647–0.856)	0.657 (65/99)	0.909 (30/33)	0.53 (35/66)
	Combined	0.866 (0.78–0.953)	0.828 (82/99)	0.758 (25/33)	0.864 (57/66)
Cross-validation (*n* = 99)	AP	0.826 (0.729–0.923)	0.808 (80/99)	0.818 (27/33)	0.803 (53/66)
	VP	0.708 (0.605–0.811)	0.677 (67/99)	0.818 (27/33)	0.606 (40/66)
	DP	0.6 (0.484–0.715)	0.535 (53/99)	0.788 (26/33)	0.409 (27/66)
	Combined	0.841 (0.747–0.936)	0.848 (84/99)	0.758 (25/33)	0.894 (59/66)
Internal validation cohort (*n* = 24)	AP	0.711 (0.489–0.933)	0.625 (15/24)	0.875 (7/8)	0.5 (8/16)
	VP	0.594* (0.339–0.848)	0.625 (15/24)	0.625 (5/8)	0.625 (10/16)
	DP	0.547** (0.257–0.837)	0.375 (9/24)	0.625 (5/8)	0.25 (4/16)
	Combined	0.789 (0.579–0.999)	0.708 (17/24)	0.625 (5/8)	0.75 (12/16)
External validation cohort (*n* = 42)	AP	0.638 (0.466–0.809)	0.524 (22/42)	0.929 (13/14)	0.321 (9/28)
	VP	0.61* (0.434–0.786)	0.595 (25/42)	0.714 (10/14)	0.536 (15/28)
	DP	0.538** (0.355–0.722)	0.405 (17/42)	0.714 (10/14)	0.25 (7/28)
	Combined	0.73 (0.563–0.896)	0.619 (26/42)	0.786 (11/14)	0.536 (15/28)

The p-value was calculated by the De Long’s test.

*p < 0.05, **p < 0.01.

AP, arterial phase; VP, venous phase; DP, delayed phase.

With the cutoff value of 0.6 that maximizes the Youden Index, the accuracy, sensitivity, and specificity of the combined model reached 0.708, 0.625, and 0.75 in the internal validation cohort and 0.619, 0.786, and 0.536 in the external validation cohort, respectively. The accuracy and the specificity of the combined model were higher or comparable than the other three single-phase models in the internal and the external validation cohorts. The sensitivity of the combined model was not lower than the other single-phase models on the internal validation cohort and the external validation cohort, except for the AP model.

**Figure 4:**
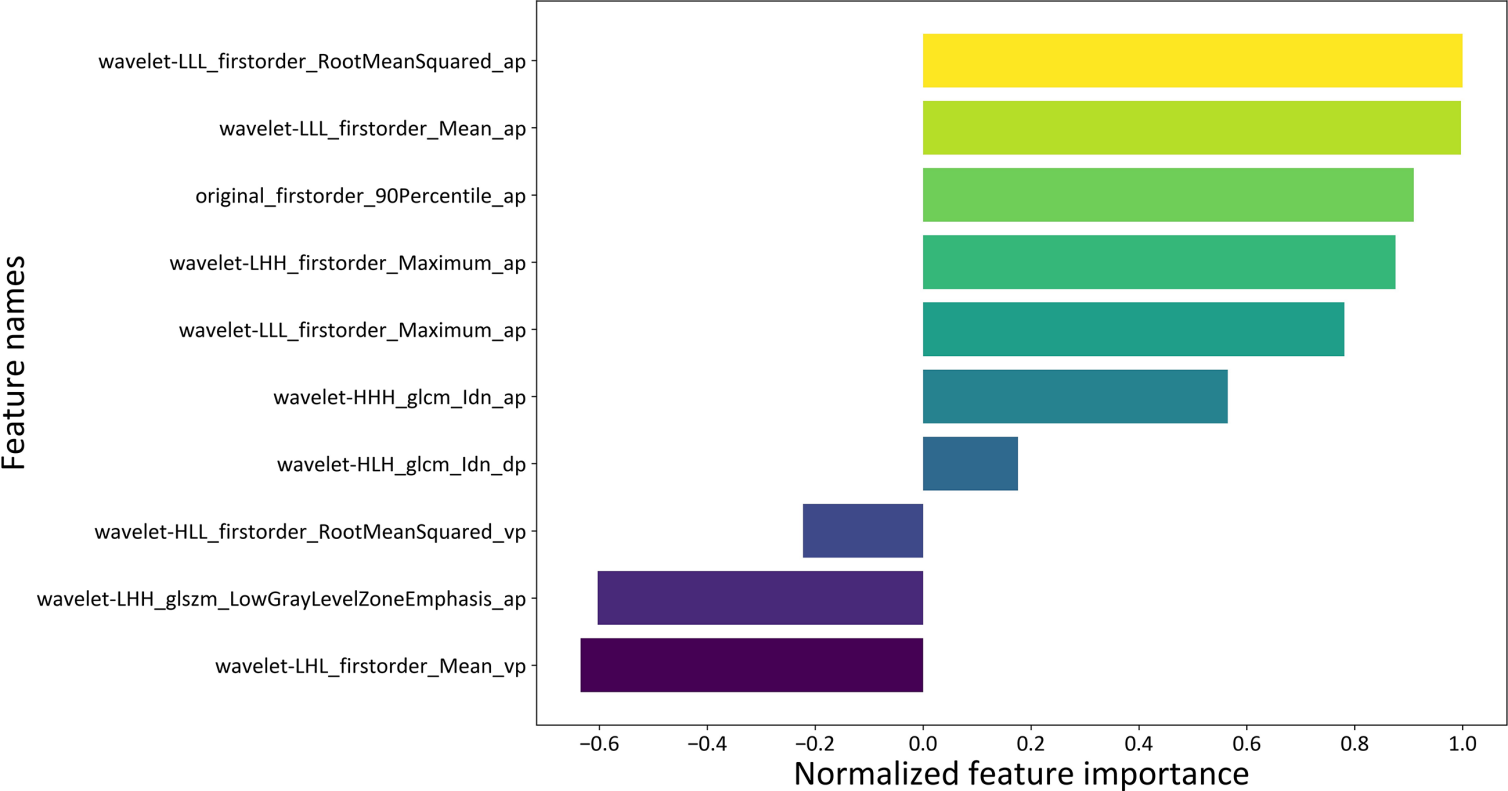
The importance of the features of the combined model.

**Figure 5:**
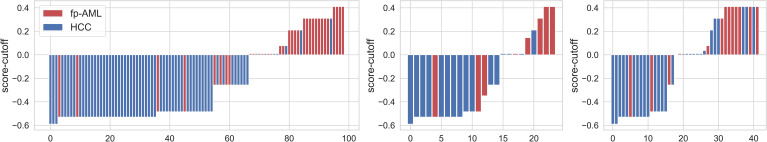
The calibrated radiomics scores for each patient in the training, internal validation, and external validation cohorts. The red bars represent the scores for fat-poor angiomyolipoma patients, while the blue bars represent the scores for the hepatocellular carcinoma patients.

The beta coefficients of the combined model were viewed as the importance of the features (illustrated in **Figure 4**), and the formula used to calculate the predicted probability of fp-AML by the combined model is listed in **Supplementary Data S1**. The features that contributed most to the diagnosis of fp-AML were wavelet-LLL_firstorder_RootMeanSquared_ap, wavelet-LLL_firstorder_Mean_ap, and original_firstorder_90Percentile_ap. On the other side, the features that contributed most to the diagnosis of HCC were wavelet-LHL_firstorder_Mean_vp, wavelet-LHH_glszm_LowGrayLevelZon-eEmphasis_ap, and wavelet-HLL_firstorder_RootMeanSquared_vp. The waterfall figure of the calibrated prediction results of each case is shown in **Figure 5**.

### Compared With the Interpretation of the Radiologists

**Figure 6:**
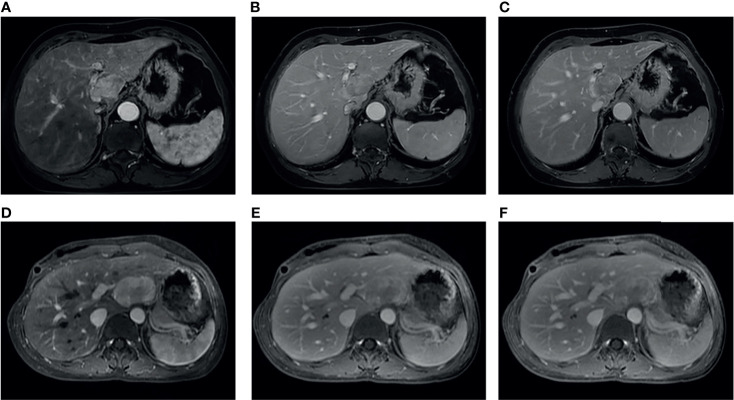
Two representative cases: case 1 **(A–C)**, a 65-year-old female without chronic hepatitis B virus (HBV) infection, and case 2 **(D–F)**, a 36-year-old female with chronic HBV infection. These two cases were both misdiagnosed as hepatocellular carcinoma by two radiologists, whereas the model output was consistent with the correct diagnosis of fat-poor angiomyolipoma.

The comparison of diagnostic performance between the models and the radiologists is shown in **Figure 3** and **Table 3**. The interobserver agreement (*κ*) values between the two radiologists were 0.565 in the internal validation cohort and 0.146 in the external validation cohort. The AUCs of radiologist 2 reached 0.594 (95%CI, 0.375–0.813) and 0.500 (95%CI, 0.351–0.649) in the internal and external validation cohorts, respectively, significantly inferior to the combined model (*p* = 0.043 and 0.027). The AUCs of radiologist 1 were 0.656 (95%CI, 0.442–0.871) and 0.643 (95%CI, 0.486–0.799) in the internal and external validation cohorts, respectively, tending to be lower than the combined model, but not significant (both *p >*0.05). In the internal and external validation cohorts, the differences in accuracy, sensitivity, and specificity between the combined model and each radiologist were not statistically significant (all *p > *0.05), except that the sensitivity of radiologist 2 was significantly lower than that of the combined model (*p* = 0.023). Representative cases in which diagnoses were corrected using the radiomics approach are shown in **Figure 6**.

**Table 3:** The detailed comparison between the performance of two radiologists and the combined model.

	AUC	Accuracy	Sensitivity	Specificity
Internal validation cohort (*n* = 24)				
Radiologist 1	0.656 (0.442–0.871)	0.667 (16/24)	0.625 (5/8)	0.688 (11/16)
Radiologist 2	0.594 (0.375–0.813)*	0.625 (15/24)	0.500 (4/8)	0.688 (11/16)
Model	0.789 (0.579–0.999)	0.708 (17/24)	0.625 (5/8)	0.750 (12/16)
External validation cohort (*n* = 42)				
Radiologist 1	0.643 (0.486–0.799)	0.690 (29/42)	0.500 (7/14)	0.786 (22/28)
Radiologist 2	0.500 (0.351–0.649)*	0.571 (24/42)	0.286 (4/14)*	0.714 (20/28)
Model	0.730 (0.563–0.896)	0.619 (26/42)	0.786 (11/14)	0.536 (15/28)

The p-value was calculated by the De Long’s test or McNemar chi-square test when appropriate.

*p < 0.05.

AUC, area under the receiver operating characteristic curve.

## Discussion

The present study showed that the combined radiomics model incorporating triple-phase CE-MRI images had a favorable predictive value for differentiating fp-AML from HCC in patients without morphological liver cirrhosis, with the AUCs of 0.866, 0.789, and 0.730, respectively, in the training cohort, internal validation cohort, and external validation cohort. The performance of the model was comparable to that of an experienced radiologist with 10 years of experience and better than that of a junior radiologist with 5 years of experience in both the internal validation cohort and the external validation cohort. To the best of our knowledge, this study represents the first multi-center and multi-scanner assessment of the role of multi-phase CE-MRI-based machine learning to differentiate fp-AML from HCC with a large sample size. The performance of this approach in the external validation cohort is encouraging, which suggests its potential to augment the diagnostic performances of radiologists, even in different centers with different scanners or different scanning parameters.

Many previous studies have used various strategies to discriminate between fp-AML and HCC. Due to the rarity of hepatic AML, especially the cases with no or minimal fat, most of these studies enrolled a small number of patients. A study with a relatively large sample size of 30 hepatic epithelioid AML indicated that specific MRI features, such as intra-tumor vessel, draining hepatic vein, prolonged enhancement, and lack of capsule, may contribute to a more confident diagnosis, consistent with the results of some previous reports (7, 27). However, some authors have put forward different views. Kim et al. (5) investigated 12 patients with lipid-poor AML and 27 patients with HCC and analyzed the presence of peripheral capsule and several imaging features related with the vascular components of AMLs on MRI images, including the feeding artery dilatation, multiple aneurysmal arteries, and the early draining veins. They found that none of these imaging features was significantly different between lipid-poor AML and HCC. The authors speculated that this could be explained by the fact that AML and HCC were both hypervascular tumors that frequently shared similar imaging features related to their vascular component and also might be attributed to the weaker arterial enhancement of gadoxetic acid and the lower spatial resolution of MRI compared to CT. In comparison to the different literatures regarding the frequency of the drainage veins on CT or MRI images in AML patients, the value varied greatly, ranging from 25 to 83.3%, according to previous reports (5, 6, 28). As to the incidence of tumor psudocapsule, this feature was reported to be found in 11.1– 42% of AMLs (3, 5). Those differences, on the one hand, could be attributed to the differences of sample sizes, patient composition, and scanning methods among these studies; on the other hand, this could also mean that the evaluation of these imaging signs is subjective and depends on the experiences of the radiologist—for example, it was pointed out that sometimes it was hard to differentiate the enhanced tumor vessels in the peripheral portion from the tumor capsules in AMLs (28). This may also explain the poor or moderate degree of agreement of the diagnosis results of the two radiologists in our study. Although having been properly trained before on the interpretation of MRI images for an accurate understanding of the useful imaging signs and following clear instructions for diagnosis in our study, radiologist 2 showed a relatively low and unsatisfactory level of sensitivity in the diagnosis in the external validation cohort due to limited diagnostic experience. Besides this, Kim et al. (5) also proposed that lipid-poor AML frequently showed more homogeneous hypointensity than HCC on the hepatobiliary phase of gadoxetic acid-enhanced MRI, and this feature could better differentiate these two diseases. However, gadoxetic acid-enhanced MRI is currently not the first-line examination of focal hepatic lesions in China due to its higher price than the conventional extracellular contrast agents and the longer scan time. Hence, compared to subjective and qualitative analyses, radiomics is objective, quantitative, and reproducible. Moreover, the radiomic analysis based on Gd-DTPA-enhanced conventional MRI images in our study does not require additional scanning time and cost, and it might prove to be a practical tool.

Our study indicated that, compared with the VP and DP models, the AP radiomics model showed a higher AUC. After adding the three phases of images together to form a combined model, the final radiomics signature contained 10 features: seven from the AP, two from the VP, and one from the DP. These results indicated that AP played a major role in distinguishing these two tumors. Although it is well known that AML and HCC usually both demonstrate intense contrast enhancement, these two tumors still seem to be different on AP. It had been proved that tumoral vessels connecting with the early draining vein in AML were more prominent and ectatic than those in HCC, and the latter tends to be faint and negligible (6). Thus, even if not showing obvious differences by visual assessment, conventional AP images might still reflect underlying, invisible, histological differences. Our results suggested that radiomics could detect these microscopic differences between fp-AML and HCC contained in routine AP images. Actually, our results were consistent with a previous study based on the measurement of mean attenuation values with 12 patients who underwent CT (28). In that study, the authors demonstrated that the hepatic AML appeared to have a more intense contrast enhancement and higher mean attenuation values exceeding 120 HU than that of HCCs on AP.

As far as we know, there is only one study based on MRI radiomics to distinguish hepatic AML from HCC. Recently, Liang et al. (7) demonstrated that the radiomics model based on AP images performed well in distinguishing epithelioid AML from HCC and focal nodular hyperplasia, especially for MRI. This was similar to our results; however, the study had not been externally verified, and the performance of the model in other participant data was not clear (26). Furthermore, unlike our study, they only used the AP images and single-layer region of interest. As mentioned above, the accuracy and specificity of the combined model were higher or comparable than the other three single-phase models in the internal and external validation cohorts in our study. Therefore, it is a better choice to combine data from multiple phases. However, whether the VOI analysis is superior to the single-layer analysis is still an unresolved question. Considering that previous studies (29, 30) had confirmed that a whole-tumor analysis had higher inter-observer consistency and better ability to reflect tumor heterogeneity than a two-dimensional analysis, we used VOI analysis in this study.

A previous study has explained the relationships between image features and texture parameters (31), which have different meanings and are expected to be related to histological features that reflect tumor heterogeneity. In our study, fp-AML was positively associated with wavelet-GLCM-inverse difference normalized on DP and AP. Interestingly, this feature reflects the local homogeneity of an image, which may be explained by the lower tissue homogeneity in HCC compared with that in fp-AMLs. Moreover, we found that HCC was significantly associated with the histogram parameters on VP, which reflected the characteristics of earlier washout on VP in HCCs (3). Besides this, GLSZM-low-gray-level-zone-emphasis (LGLZE) on AP was one of the top three ranked parameters for predicting HCC in our study. The GLSZM provides information on the size of the homogeneous zones for each gray level in three dimensions, and LGLZE is the distribution of the low gray level zones. According to a previous study, compared to high gray-level values, gray level runs with low gray-level values in two-dimensional images of the cell nuclei in ovarian cancer patients, indicating a higher probability for strong invasion ability and a poor prognosis (32), which seemed to be in agreement with our results.

There are several limitations in our study. Firstly, compared with HCC, fp-AML is encountered less frequently in clinical practice owing to its rarity. Although we performed a multicentric trial employing a relatively larger sample size, the number of patients with fp-AML was still far less than the patients with HCC. However, our study of 55 patients represents, to our knowledge, the largest cohort of hepatic fp-AML patients analyzed for differential diagnosis of HCC so far. In addition, we followed a fp-AML–HCC ratio of 1:2 to lessen the impact of imbalanced datasets that exist. Secondly, using multicentric CE-MRI datasets for radiomic feature extraction can pose a greater challenge due to the variations resulting from differences in imaging equipment and acquisition parameters. To overcome this problem, we adopted the histogram matching techniques to correct scanner-dependent intensity variations. Besides this, it has been proved that if the spatial resolution of the MRI images used in radiomics analysis is high enough, it can offset the influence of different scan parameters on the results (33). In our study, all three centers adopted three-dimensional fs T1W gradient-echo sequence for dynamic enhancement imaging, which provided high-slice selective spatial resolution (2 to 3 mm) (34). Thirdly, radiomics signature was constructed using CE-MRI images only in this multicenter study. The reason was that the CE-MRI images were retrospectively collected, so we finally adopted only the enhanced sequence to obtain the largest possible sample size.

In conclusion, this multicenter study indicates the proposed CE-MRI-based radiomics model incorporating triple-phase images that can be useful for differentiating between fp-AML and HCC and yields comparable or better performance than that of two radiologists in both the internal validation cohort and the external validation cohort.

## Data Availability Statement

The raw data supporting the conclusions of this article will be made available by the authors, without undue reservation.

## Ethics Statement

The studies involving human participants were reviewed and approved by the institutional review board of Zhongshan Hospital, Guangdong Sun Yat-Sen University Cancer Center, Guangdong Provincial People’s Hospital. Written informed consent for participation was not required for this study in accordance with the national legislation and the institutional requirements.

## Author Contributions

YKZ and XZ carried out the studies, participated in collecting data, and drafted the manuscript. YZ and MW carried out the studies and participated in collecting data. LH participated in collecting data. LM, YY, and XL performed the statistical analysis. MW, MZ, and ZL participated in its design. All authors contributed to the article and approved the submitted version.

## Funding

The authors declare that this study received funding from the Key R&D Program of Guangdong Province, China (2021B0101420006), the National Science Fund for Distinguished Young Scholars (81925023), the National Natural Science Foundation of China (82102145), the Shanghai Municipal Key Clinical Specialty (shslczdzk03202) and the Guangdong Medical Research Fund (A2021086). The funders were not involved in the study design, collection, analysis, and interpretation of data, the writing of this article, or the decision to submit it for publication.

## Conflict of Interest

YY, LM, and XL were employed by the company Deepwise AI Lab, Deepwise Inc.

The remaining authors declare that the research was conducted in the absence of any commercial or financial relationships that could be construed as a potential conflict of interest.

## Publisher’s Note

All claims expressed in this article are solely those of the authors and do not necessarily represent those of their affiliated organizations, or those of the publisher, the editors and the reviewers. Any product that may be evaluated in this article, or claim that may be made by its manufacturer, is not guaranteed or endorsed by the publisher.

## Abbreviations

CE, contrast-enhanced; MRI, magnetic resonance imaging; AML, angiomyolipoma; fp, fat-poor; HCC, hepatocellular carcinoma; VOI, volume of interest; AP, arterial phase; VP, venous phase; DP, delayed phase; AUC, area under the receiver operating characteristic curve; PEComas, perivascular epithelioid cell tumors; CT, computed tomography; HBV, hepatitis B virus; HCV, hepatitis C virus; FNAC, fine needle aspiration cytology; Gd‐DTPA, gadolinium-diethylene triamine pentaacetic acid; T1W, T1-weighted; FS, fat-saturation; T2W, T2-weighted; LI-RADS, liver imaging reporting and data system; MITK, medical imaging interaction toolkit; ICC, inter- and intra- class correlation coefficient; LoG, Laplacian of Gaussian; GLCM, gray-level co-occurrence matrix; GLSZM, gray-level size zone matrix; GLRLM, gray-level run length matrix; GLDM, gray-level dependence matrix; JMIM, joint mutual information maximization; LR, logistic regression; ROC, receiver operating characteristic; IDN, inverse difference normalized; LGLZE, low gray-level zone emphasis.
