# Supplementary material for: Radiomics Based on Contrast-Enhanced MRI in Differentiation Between Fat-Poor Angiomyolipoma and Hepatocellular Carcinoma in Noncirrhotic Liver: A Multicenter Analysis
Source: Front Oncol. 2021 Oct 13;11:744756. doi: 10.3389/fonc.2021.744756 (PMC8548657; doi:10.3389/fonc.2021.744756)
Supplement: Supplementary file 1 [file DataSheet_1.docx]

Supplementary Material

# Supplementary Data

The predicted probability of fat-poor hepatic angiomyolipomas by the combined model was calculated using the following formula:

$$probability= \frac{1}{1+e^{-y}}$$

y=0.00331*original_firstorder_90Percentile_ap

+0.00318*wavelet-LHH_firstorder_Maximum_ap

+-0.00219*waveletLHH_glszm_LowGrayLevelZoneEmphasis_ap

+0.00205*wavelet-HHH_glcm_Idn_ap

+0.00284*wavelet-LLL_firstorder_Maximum_ap

+0.00362*wavelet-LLL_firstorder_Mean_ap

+0.00364*wavelet-LLL_firstorder_RootMeanSquared_ap

+0.00064*wavelet-HLH_glcm_Idn_dp

+-0.00231*wavelet-LHL_firstorder_Mean_vp

+-0.00081*wavelet-HLL_firstorder_RootMeanSquared_vp
